# Cigarette Smoking and its Relationship with Perceived Familial Support and Religiosity of University Students in Tabriz

**Published:** 2015-06

**Authors:** Hamid Allahverdipour, Abbas Abbasi-Ghahramanloo, Asghar Mohammadpoorasl, Pouran Nowzari

**Affiliations:** 1 Department of Health Education & Promotion, School of Health & Nutrition, Tabriz University of Medical Sciences, Tabriz, Iran; 2 Department of Epidemiology, Iran University of Medical Science, Tehran, Iran; 3 Tabriz Health Services Management Research Center,Tabriz University of Medical Sciences, Tabriz, Iran; 4 Evaluator, Health Education and Nutrition Consultant. 2874 E. Niles Ave, Fresno, CA 93720,USA

**Keywords:** *Cigarette Smoking*, *Perceived Familial Support*, *Religiosity*, *Risk Taking behaviors*, *Students*

## Abstract

**Objective:** The goal of the present study was to assess the prevalence of cigarette smoking and its relationship to other risk taking behaviors, perceived familial support and religiosity among college students in Tabriz, Iran.

**Method:** In this study, 1837 randomly selected students participated and completed a self-administered questionnaire inquiring demographic characteristics, risk taking behaviors, Aneshensel and Sucoff’s 13-items one-dimensional perceived Parental support scale and 28 - items Kendler’s general religiosity scale.

**Results**: In general, 15.8 % of the students were cigarette smokers. The results indicated that being male (OR = 3.21), living alone or with friends (OR = 2.00), having a part-time job (OR = 1.98), alcohol consumption during the past 30 days (OR = 3.67), hookah use (OR = 5.23), substance abuse (OR = 1.69), familial support (OR = 0.97) and religiosity (OR = 0.98) have statistically significant relationships with cigarette smoking.

**Conclusion:** Our study represents the co-occurrence of risky behaviors. Cultural context in the traditional communities seems to show the crucial role of familial support and religiosity traits with the female gender as predictive factors to not smoke cigarette and perform other risky behaviors.

It is clear that smoking harms nearly every organ of the body, causing many diseases and reducing the health of smokers in general (1). It is estimated that more than 5 million of the world population die of smoking each year; many of these death cases occur in countries with low or medium income (2).The prevalence of cigarette smoking has been reported as 20.8% in the United States (3), 22% in United Kingdom and 17.5% in Iran (2). The prevalence of cigarette smoking among the university students has been reported to be 8.6% to 28.6% in many studies in several countries (4-8). Such a broad range of prevalence is primarily due to the variety of the definitions of smoking and the location where such studies have taken place. Smoking is a complex and multifactorial behaviour, and numerous studies have linked it to genetic predisposition, demographic and psychosocial factors, tobacco policy, accessibility of cigarettes, social values and norms regarding tobacco smoking, role models, academic performance and religious beliefs (3, 9-14). 

A great deal of research has focused on the identification of factors that elevate or reduce the risk of engaging in substance use as well as cigarette use and other health risk behaviors among young people(15-17). The status of parental support and religiosity are known as predictive factors in the onset of substance use among adolescents. Several studies conducted on Christians reported that low prevalence of cigarette smoking is seen in people with stronger religious beliefs (18-21).

Religious beliefs, as one of the individual traits, provides a unifying framework for interpreting, identifying meaning in, and coping with difficult life experiences as well as adapting healthy behaviors during adulthood independent of the type of religion (22). Numerous studies conducted in different societies indicated that religious individuals significantly demonstrate a range of healthy behaviors (23, 24). Additionally, some studies showed indirect effects of religiosity on the health status of individuals through promoting positive health behaviors like healthy eating, physical activity and modifying and/or preventing unhealthy behaviors like smoking and drug use(15, 22).

Parental support is another factor that protects adolescents against risky behaviours. Evidence shows that adolescents from more cohesive families are less likely to smoke or use substances (25-27). Parental positive influence on the cessation of adolescent smoking behaviours is reported in numerous studies (28, 29). Conversely, loneliness and social isolation are reported as predictors of engaging in high risk behaviours(30).

Conducting further studies on cigarette smoking among college students is necessary as the high smoking rate among the students can be a convenient indicator of risk taking behavior such as cigarette smoking among the young people (31). We should also further explore the role that cultural context, as a determining factor, has on how familial support and religious beliefs are perceived by young people. This study sought to expand the understanding of factors related to cigarette smoking behaviours among Iranian college students in the city of Tabriz by focusing on the relationship between the risk taking behaviours and students' perceived familial support and their religiosity.

## Material and Methods

*Participants and Procedure*

This study was part of a larger project conducted on 1837 university students who were randomly selected to participate in the research from April to May 2011 in Tabriz, Iran. First, three faculties were randomly selected from each of the nine universities in Tabriz. Then, based on the number of students in each university and the selected faculties, a proportional number of students (n = 2128) were selected randomly using cluster sampling from each of the selected faculties. A consent form was developed by the researchers and was reviewed and approved by the Institutional Review Board (IRB) at Tabriz University of Medical Sciences. Finally, all 2128 randomly selected students were contacted to be recruited as the study population. A total of 1837 (86.1%) signed the IRB approved consent form and voluntarily agreed to participate in the study.

*Measures*

A survey instrument was developed to collect demographic data as well as data related to the study variables. A pilot study was conducted to assess the reliability and validity of the survey instrument. The pilot study was administered with 97 participants who were university students with similar characteristics as the participants in the main study. The pilot study provided input about the clarity, length, comprehensiveness and the average time needed to complete the survey, as well as collecting data to estimate the internal consistency of the survey instrument. The survey tool was revised using the input provided by the pilot study. 

*Demographics*

Background data were collected on age, gender, marital status, residency status (native, non-native), living arrangement (living with parents, dormitory, alone or shared home), employment, hookah use (at least one session per month), alcohol use in the past 30 days, history of substance use and having unsafe sex (i.e., using drugs or alcohol before the last sexual relationship, having sexual intercourse with numerous persons, or having sex without using condom).

*Cigarette Smoking*

To assess whether or not the participants had smoking history, we used their responses to the following question: “Have you ever smoked a cigarette?" The next smoking question focused on the frequency of smoking. Students who smoked 100 cigarettes or more in their lifetime irrespective of their current smoking status were considered as smokers.

*Parental Support*

Participants were asked to complete Aneshensel and Sucoff’s 13-items one-dimensional Parental support scale (32) to assess the relationship between students and their parents or guardians. The scale was translated into Farsi by Farhadinassab et al. (15). Example of scale items included, "My mother and/or father show(s) me if I can trust them/him/her," and "really understand(s) me". The participants were asked to choose one of the following responses: "1) strongly agree, 2) agree, 3) disagree, 4) strongly agree, 5) I do not know". One item on the scale, "My mother and/ or father don't/doesn't pay enough attention to me," was coded inversely. The scores of this test ranged from 13 to 65, with higher scores indicating higher parental support. The internal consistency of the scale was 0.86.

*General Religiosity*

General religiosity of the students was measured by a modified set of 28 items based on Kendler’s general religiosity scale (33) , and was also adopted to Farsi by Farhadinassab et al. (15) . Examples of the items were as follows: 1) "I ask God to help me make important decisions;"2) "I feel God’s love for me, directly or through others;" and 3) "Every day I see evidence that God is active in the world". Response categories were coded from 1 to 5 (strongly disagree, disagree, neutral, agree and strongly agree, respectively). The scores of this test ranged from 28 to 140, with higher scores indicating higher religious beliefs. An estimated reliability coefficient (α = 0.97) indicated that the measure of general religiosity was internally consistent.

*Data Analysis*

Chi-square test was used to assess the relationship of qualitative variables with smoking status; independent t-test was performed to assess the relationship of qualitative variables with smoking status; and Logistic regression model was used to measure all related factors in 0.2 alpha level in univariate analyses. All analyses were performed using SPSS-16 (SPSS Inc., Chicago, Illinois, USA); and an alpha level of .05 was considered statistically significant. 

## Results

The age range of the study participants was 18 to 34 with the mean age of the 22.09 years. The participants included 736 males (40.1%) and 1101 females (59.9%); of the total of 1837 students, 287 (15.6%) were cigarette smokers. With respect to living arrangement, nearly 38.7 % of the respondents reported that they were living with their family/parents in their hometown, 970 (53.4%) were living in dormitory, 203 (11.7%) in a shared home or alone. Of the participants, 144 (7.8%) were married. Concerning the history of high risk behaviors, the findings revealed that 147 (8.0%) of the participants reported having a history of alcohol use in the past 30 days, 141 (7.7%) had a history of long life substance use, 156 (8.5%) had a history of hookah use. In addition, 198 (10.7%) respondents reported to have a history of having unsafe sex. 

Cigarette smoking is more prevalent in male students. After considering cigarette smoking status as a dichotomous variable, the prevalence of cigarette smoking amongst all students was 15.8% (CI 95%: 14.2-17.5) with 28.5% (CI 95%: 25.4-31.9) for male and 7.1% (CI 95%: 5.7-8.8) for female students. Table 1 illustrates the univariate analysis results of determining the relationship between cigarette smoking and independent variables. This indicates that all the following variables including being single, male, non- native, living alone or with friends, part-time job, alcohol consumption, hookah use, substance abuse and sexual risk-taking behavior have a statistically significant relationship with cigarette consumption (all p < .05).

Independent t- test was performed to assess the relationship between the students' perceived familial support as well as their religiosity with cigarette smoking status. The mean of perceived familial support score for smoker and non-smoker students were 50.3±9.5 and 42.3±12.1, respectively (P<0.001). The mean of religious beliefs score for non-smoker and smoker students were 113.2±16.0 and 97.1±20.9, respectively (P<0.001).

All variables were entered into the logistic regression model for multiple analyses of the factors relating to cigarette smoking status. 

The analysis results indicated that male gender (OR = 3.21), living alone or with friends (OR=2.00), part-time job while going to school (OR = 1.98), alcohol consumption during the past 30 days (OR = 3.67), hookah use (OR = 5.23), substance abuse (OR = 1.69), perceived family support score (OR = 0.97) and religiosity score (OR = 0.98) have a statistically significant relationship with cigarette smoking behavior of the students (Table 2).

**Table1:** Cigarette smoking relationship to demographic factors and high risk behaviors of students

**Characteristics**	**Nonsmoker** **n (%)**	**Smoker** * **n (%)**	**Overall** **n (%)**	**p-value**
**Gender**				
Male	526(71.5)	210(28.5)	736(40.1)	P<0.001
Female	1023(92.9)	78(7.1)	1101(59.9)	
**Marital status**				
Single	1417(83.8)	273(16.2)	1690(92.2)	P=0.111
Married	128(88.9)	16(11.1)	144(7.8)	
**Residency status**				
Native	613(87.0)	92(13.0)	705(38.7)	0.015
Non-native	922(82.7)	193(17.3)	1115(61.3)	
**Living in **				
Parental home	562(87.4)	81(12.6)	643(35.4)	P<0.001
Dormitory	823(84.8)	147(15.2)	970(53.4)	
Single house	146(71.9)	57(28.1)	203(11.2)	
**Having job**				
Yes	159(69.7)	69(30.3)	228(12.4)	P<0.001
No	1392(86.6)	216(13.4)	1608(87.6)	
**Alcohol use (past 30 days)**				
No	1485(88.2)	198(11.8)	1683(92.0)	P<0.001
Yes	56(38.1)	91(61.9)	147(8.0)	
**Hookah use(at least once per month)**				
No	1492(88.6)	192(11.4)	1684(91.5)	P<0.001
Yes	57(36.5)	99(63.5)	156(8.5)	
**Substance abuse(ever use)**				
No	1477(87.0)	220(13.0)	1697(92.3)	P<0.001
Yes	73(51.8)	68(48.2)	141(7.7)	
**Having unsafe sex**				
No	1419(86.3)	226(13.7)	1645(89.3)	P<0.001
Yes	133(67.2)	65(32.8)	198(10.7)	

* Smokers: respondents who indicated smoking 100 cigarettes or more in their lifetime irrespective of current smoking status.

**Table 2:** Logistic regression analysis of the relationship between smoking status and risk variables in a sample of Iranian students in Tabriz (2011)

Variables	OR	CI 95 %	P-value
Gender(male)	3.21	2.31-4.45	<0.001
Marital status(being single)	1.51	0.74-3.10	0.259
Residency status(non-aboriginal)	1.04	0.91-1.97	0.291
Living in Parental home	1	-	-
Dormitory	1.09	0.76-1.56	0.645
Single house	2.00	1.23-3.25	0.005
Working along with education	1.98	1.32-2.97	0.001
Alcohol use in the past 30 days	3.67	2.26-5.97	<0.001
Hookah use(at least once per month)	5.23	3.38-8.09	<0.001
Substance abuse(ever)	1.69	1.02-2.82	0.043
Having unsafe sex	0.72	0.44-1.18	0.190
Age(higher)	1.04	0.97-1.12	0.242
Higher score of family support	0.97	0.96-0.99	0.001
Higher score of religious beliefs	0.98	0.97-0.99	<0.001

Finally, bivariate correlation analysis between perceived familial support, religiosity and age revealed a significant relationship between perceived familial support and religiosity (r = 0.48; P-value < 0.001), and between perceived familial support and age (r = -0.07; p-value = 0.002). However, no significant relationship was found between religiosity and age (r = 0.04, P-value = 0.074).

## Discussion

In this cross-sectional study, an association between cigarette smoking and perceived familial support and religiosity in young adults has been demonstrated. According to data analyses, lack of perceived familial support has a correlation with cigarette smoking in young people who are at high risk for cigarette smoking. In addition, students living in the single or shared homes reported a more significant history of cigarette smoking than their counterparts living in dormitories or with their parents. Moreover, our findings confirmed that the level of familial support may reduce the probability of cigarette smoking among young adults, and this is consistent with findings of previous studies (34, 35). Numerous studies have argued that parental protective role, parental control and monitoring have a strong potential to protect young adults against substance use as well as other deviant behaviors (34, 36, 37). Parental monitoring as a way of transmitting behavioral norms to children is proved effective during the later years of life when there is a supportive connectedness between parents and young adults. In other words, the quality of parent-child relationship may also play a preventive role against high risk behaviors through parental support, or a feeling that their parents will take care of them in later years (38). Additionally, evidences indicate that the acceptance of parents' values with a positive attitude may also be associated with lower tendency to smoking, drinking alcohol or using drugs amongst young people (39, 40).

Our data also revealed that the existing religiosity belief is another factor that may act as a preventive factor against cigarette smoking among the young. As mentioned, an inverse significant relationship was found between religiosity of the participants and their smoking related behaviors. The findings of the present study are consistent with those of several other studies that have demonstrated the role of religiosity and religious beliefs in the prevention of high risk behaviors as well as a tobacco use (15, 16, 22). Additionally, the results of a meta-analysis confirmed the protective effects of religiosity on substances use (41). Gillum (42) declared that although religiosity, like education, has been reported to be inversely associated with cigarette smoking, religious people are more likely to under report smoking leading to spurious claims. In other words, religiosity plays an important and critical role in preventing young adults from cigarette and substance use by conveying to them the religion's expectations about healthy lifestyle, engaging in healthy behaviors and refusing high risk behaviors (43-45) that would help promote healthy life styles at the individual and community levels. 

Additionally, the results of this study revealed that the prevalence of cigarette smoking is higher among male than female students. Different results have been obtained in other studies. For example, in a study conducted in Brazil, 16% of the students had smoked within 30 days prior to the study with no statistically significant difference between the male and female students (46). In another study conducted in Hungary, the percentage of cigarette smoking by young boys and girls was reported to be 41.8% and 38.8%, respectively, showing no significant difference between the two sexes (9). However, studies show that substance abuse, as well as other high risk behaviors, happen less among girls (47-49) though these differences in findings among various studies may be attributed to the differences in definition of smoking. Moreover, single students had higher incidences of smoking than the married. Also, in a study conducted on students in Iran, the prevalence of cigarette smoking among single and married students was 22.8% and 10.1%, respectively, showing a statistically significant difference among the two groups, and this is consistent with our findings (50). 

Our findings were consistent with previous findings in the literature with respect to the relationship between high risk behaviors (i.e., smoking, drinking and unsafe sexual behaviors) and cigarette smoking. They were also consistent with a study carried out in the USA (51) which showed a significant relationship between cigarette smoking and age of the first sexual experience. 

## Limitations

Several aspects of this study can limit the application of the findings: first, the cross-sectional nature of the study serves only as an evidence for the relationship between independent variables and cigarette smoking status and does not show causality. Second, despite using a well-designed methodology and sampling method, generalization of the results is limited only to the students in the city of Tabriz. Finally, confounder variables such as psychiatric disorders were not considered in the students’ families.

## Conclusion

Cigarette smoking and its co-occurrence with other high-risk behaviors in this group of young people is indicative of an alarming rate of spreading the prevalence of unhealthy behaviors among students. According to the results of this study, the variables of familial support and religiosity can serve as preventive factors in cigarette smoking as well as other risk taking behaviors. Consequently, focusing on familial support and religious beliefs can be considered as elements of effective preventative programs which will help design and develop comprehensive health education programs with the goal of empowering the individuals and the community.
